# Synthesis of High-Molecular-Weight Branched Polyethylene Using a Hybrid “Sandwich” Pyridine-Imine Ni(II) Catalyst

**DOI:** 10.3389/fchem.2022.886888

**Published:** 2022-05-04

**Authors:** You Ge, Qi Cai, Yuyin Wang, Jiangang Gao, Yue Chi, Shengyu Dai

**Affiliations:** ^1^ School of Chemical and Environmental Engineering, Anhui Polytechnic University, Wuhu, China; ^2^ Institutes of Physical Science and Information Technology, Key Laboratory of Structure and Functional Regulation of Hybrid Materials of Ministry of Education, Anhui University, Hefei, China; ^3^ Key Laboratory of Advanced Structural Materials of Ministry of Education, College of Material Science and Engineering, College of Chemistry and Life Science, Advanced Institute of Materials Science, Changchun University of Technology, Changchun, China

**Keywords:** hybrid “sandwich”, Ni(II) and Pd(II) catalysts, highly branched, high-molecular-weight, pyridine-imine

## Abstract

Most pyridine-imine Ni(II) and Pd(II) catalysts tend to yield low-molecular-weight polyethylene and ethylene-based copolymers in olefin insertion polymerization, as the unilateral axial steric structure of such complexes often cannot provide effective shielding of the metal center. In this study, we synthesized a series of hybrid “semi-sandwich” and “sandwich” type pyridine-imine Ni(II) complexes by incorporating diarylmethyl or dibenzosuberyl groups onto 8-aryl-naphthyl motif. The as-prepared Ni(II) complexes afforded highly branched polyethylene with high molecular weights (level of 10^5^ g/mol), and moderate activities (level of 10^5^ g/(molh)) in ethylene polymerization. Most interestingly, compared to “semi-sandwich” Ni(II) complexes bearing (2-diarylmethyl-8-aryl)naphthyl units, the “full-sandwich” counterpart containing (2-dibenzosuberyl-8-aryl)naphthyl motif was able to produce higher-molecular-weight polyethylene with higher branching density. In addition, the effect of remote non-conjugated electronic substituents in diarylmethyl groups of the Ni(II) system was also observed in ethylene polymerization.

## Introduction

**CHART 1:**
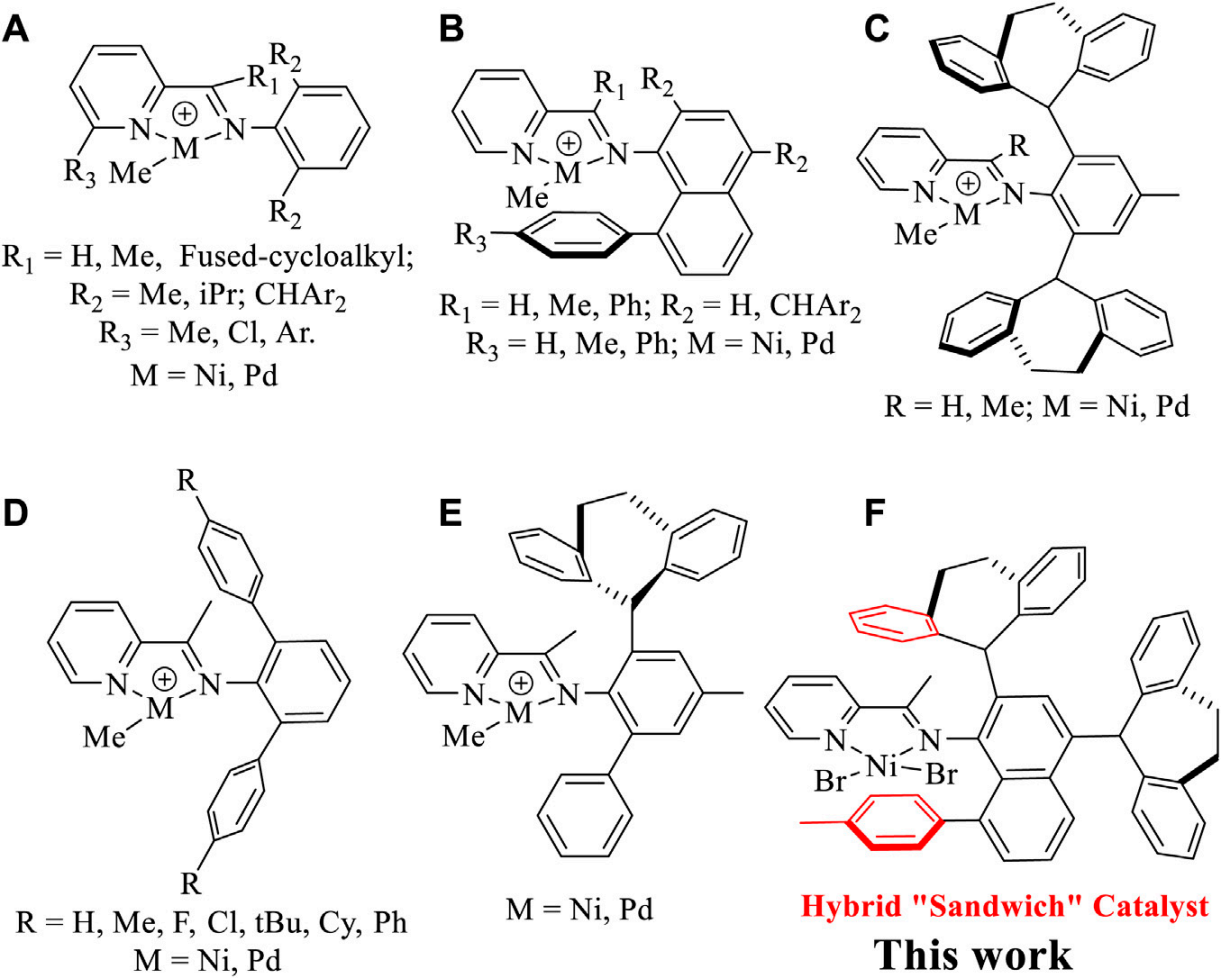
Modifications of pyridine-imine Ni(II) and Pd(II) catalysts **(A–E)**, and our current work **(F)**.

As known, the [N, N] bidentate α-diimine Ni(II) and Pd(II) complexes bearing double-sided axial steric structures represent a mainstream catalytic system, which tend to yield high-molecular-weight polyethylene and ethylene-polar monomer copolymers. (Dai et al., 2016a; Gong et al., 2019a; Guo et al., 2018; Meinhard et al., 2007; Rhinehart et al., 2013; Dai et al., 2015; Xia et al., 2020; Zhao et al., 2021; Wang et al., 2020a; Zhang et al., 2013; Gong et al., 2019b; Liao et al., 2019; Zhong et al., 2017a; Zhong et al., 2017b; Zhong et al., 2019; Abedini et al., 2021; Kanai et al., 2019; Zhang et al., 2020; Ma et al., 2021; Li et al., 2021a; Allen et al., 2015). In contrast, pyridine-imine Ni(II) and Pd(II) catalysts often give rise to low-molecular-weight oligomers because the only unilateral axial steric hindrance rising from the imine motif hardly shields the metal well in most cases. (Dai et al., 2016b). Consequently, strategies that are effective in suppressing the chain transfer to bring forth high-molecular-weight products in α-diimine systems are often not applicable to the pyridine-imine systems. For example, by using the bulky diarylmethyl anilines, the α-diimine Ni(II) and Pd(II) catalysts can generate high-molecular-weight and even ultra-high-molecular-weight polyethylenes. (Rhinehart et al., 2013; Dai et al., 2015; Zhao et al., 2021; Gong et al., 2019b). However, the pyridine-imine system derived from same diarylmethyl anilines provides only branched ethylene oligomers. (Chen et al., 2018; Li et al., 2021b; Yan et al., 2021; Wang et al., 2020b; Guo et al., 2019). In fact, since Laine et al. reported that the first example of pyridine-imine nickel-catalyzed ethylene polymerization yielded low-molecular-weight branched polyethylene, (Laine et al., 1999), many attempts, including the steric tuning of the *o*-aryl substituents, modifying the pyridine backbone and adjusting ligand electronic effect have been made to improve this situation, but no visible improvement was achieved (Chart 1A). (Bianchini et al., 2010; Laine et al., 2000; Meneghetti et al., 1999; Huang et al., 2016; Sun et al., 2015a; Huang et al., 2015; Sun et al., 2015b; Yue et al., 2014; Sun et al., 2012; Chen et al., 2016) Recently, we simultaneously integrated 8-aryl-naphthyl and dibenzhydryl substituents into the pyridine-imine system, making the resultant complexes able to effectively suppress chain transfer in the ethylene polymerization, thus yielding high molecular weight polyethylene (*M*_n_ well above 100 kg/mol) (Chart 1B). (Dai et al., 2016b) More recently, the dibenzosuberyl groups were also employed in the pyridine-imine system to enhance the polyethylene molecular weight (*M*_n_ up to 124 kg/mol) via a rotation-restricted strategy (Chart 1C). (Peng et al., 2021; Li and Dai, 2021) In contrast, the pyridine-imine consisting of N-terphenyl structure failed to retard chain transfer, thus only hyperbranched ethylene oligomers and ethylene-methyl acrylate (MA) co-oligomers can be obtained (Chart 1D). (Fan et al., 2021; Yan et al., 2022; Fan et al., 2022) In addition, the effectiveness of a single dibenzosuberyl group in unsymmetrical iminopyridyl Ni(II) and Pd(II) catalysts in retarding the chain transfer was also demonstrated (Chart 1E). (Ge et al., 2021) In this study, the dibenzosuberyl and 8-aryl-naphthyl units are integrated into the pyridine-imine nickel catalyst at the same time and the resulting hybrid “sandwich” catalyst is capable of catalyzing ethylene polymerization to yield highly branched polyethylene with high molecular weights (Chart 1F).

## Results and Discussion

### Synthesis and Characterization of Pyridine-Imine Nickel and Palladium Complexes

**SCHEME 1:**
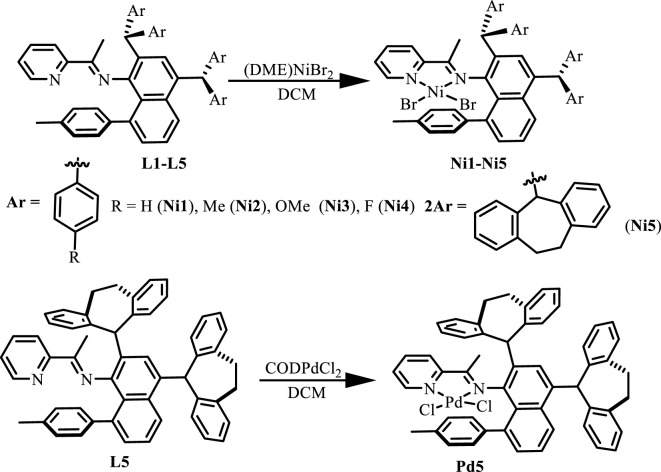
Synthesis of “semi-sandwich” and “sandwich” type pyridine-imine Ni(II) (**Ni1**-**Ni5**) and Pd(II) (**Pd5**) complexes.

**FIGURE 1:**
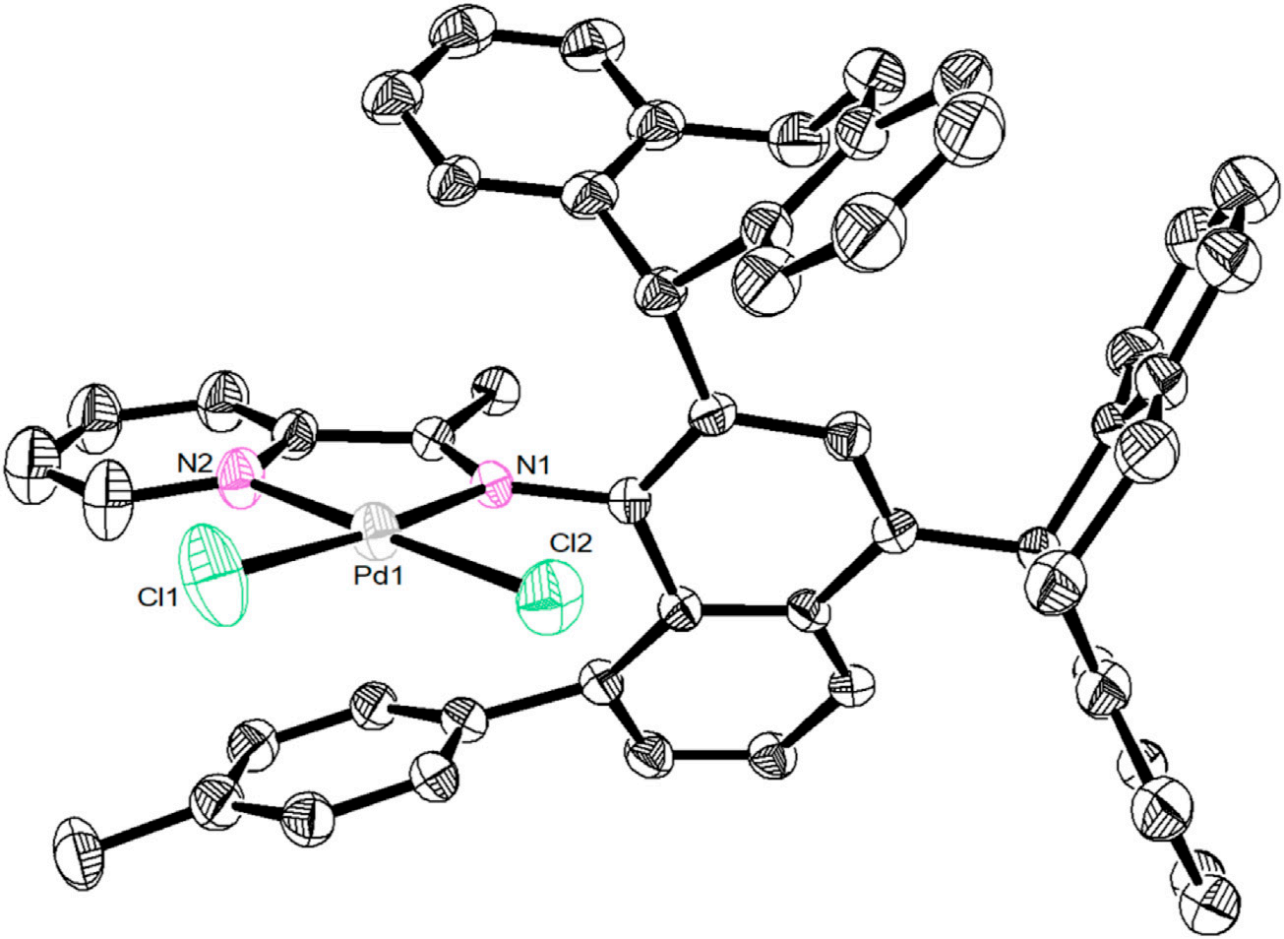
Solid-state molecular structure of **Pd5** (2150684) at the 30% probability level. All solvent molecules and hydrogen atoms are omitted for better clarity.

**FIGURE 2:**
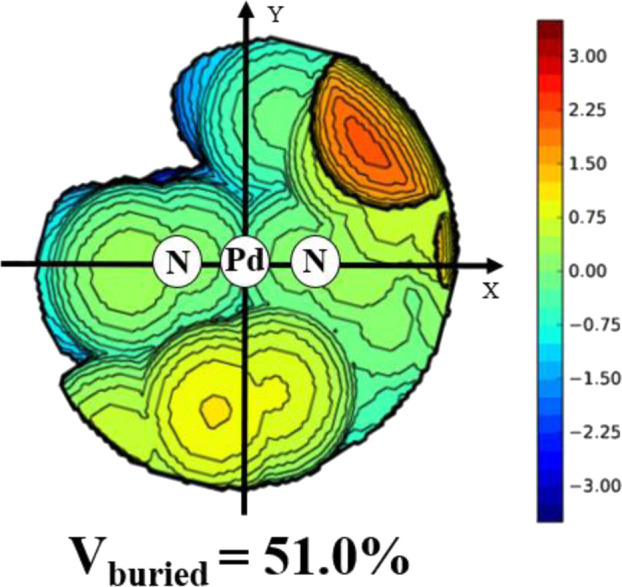
Topographic steric maps of complex **Pd5**.

Ligands **L1-L5** were synthesized according to the previous reports. (Li and Dai, 2021). Treating these ligands with 1.0 equiv. of [NiBr_2_(DME)] (DME = Dimethoxyethane) in dichloromethane at ambient temperature yielded Ni(II) complexes **Ni1-Ni5** in excellent yields (81–94%) (Scheme 1). The purity and identity of **Ni1-Ni5** were examined by elemental analysis and MALDI-TOF MS (Supplementary Figures S3–7). Similarly, the Pd(II) complex **Pd5** was synthesized by exposing the ligand **L5** to [PdClMe(COD)] (COD = 1, 5-cyclooctadiene) in dichloromethane at ambient temperature (Scheme 1). The obtained Pd(II) complex was verified by ^1^H and ^13^C NMR (Supplementary Figures S1, 2), ESI-MS (Supplementary Figure S8), and elemental analysis. The single crystal **Pd5** was obtained by layering its CH_2_Cl_2_ solution with diethyl ether at ambient temperature (Figure 1). The complex **Pd5** displays an approximate planar square geometry at the Pd(II) center, and the 4-methylphenyl group and phenyl ring of dibenzosuberyl substituent lie nearly parallel to the five-membered chelate ring and effectively block the axial coordination site of the Pd(II) complex, which is responsible for the retardation of the undesired chain transfer. Here, we also provide the buried volume diagram of **Pd5** complex analyzed by SambVca 2.0 program (Figure 2). (Falivene et al., 2015) As expected, the complex **Pd5** possessed crowded environment around the palladium center with the percent buried volume of 51.0%. This type of bulky substituents helps to suppress chain transfer during polymerization to obtain high molecular weight polymers. (Deng et al., 1997; Talarico et al., 2004).

### Ethylene Polymerization

**FIGURE 3:**
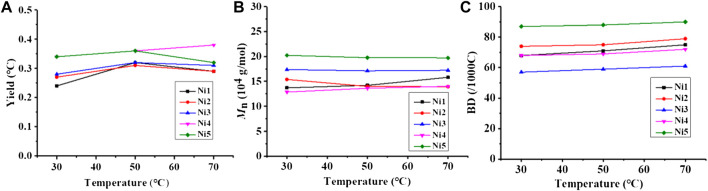
Comparisons on yield **(A)**, molecular weight **(B)**, and branching density **(C)** of polyethylene yielded with catalysts Ni1-Ni5 at 30–70°C.

**FIGURE 4:**
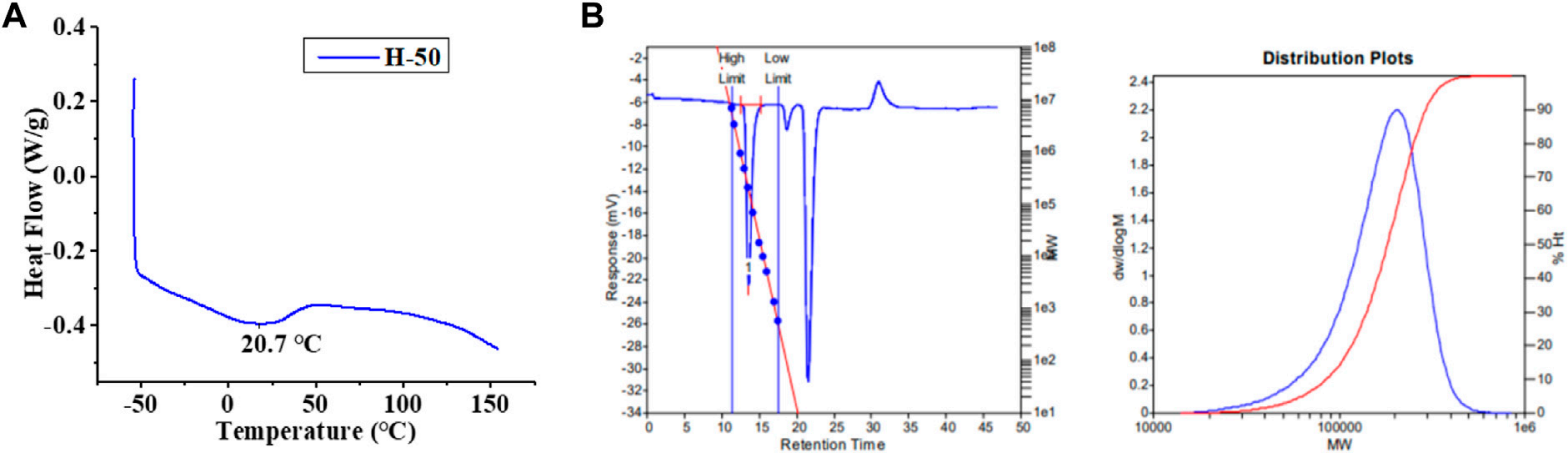
DSC **(A)** and GPC **(B)** of the branched polyethylene obtained by using Ni1 at 50°C (Table 1, entry 2).

**FIGURE 5:**
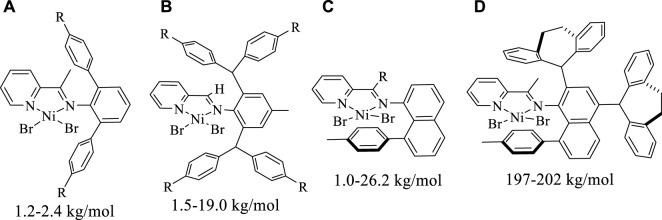
Comparisons on molecular weights of polyethylene yielded with previously reported nickel catalysts **(A–C)** and Ni5 **(D)**.

**FIGURE 6:**
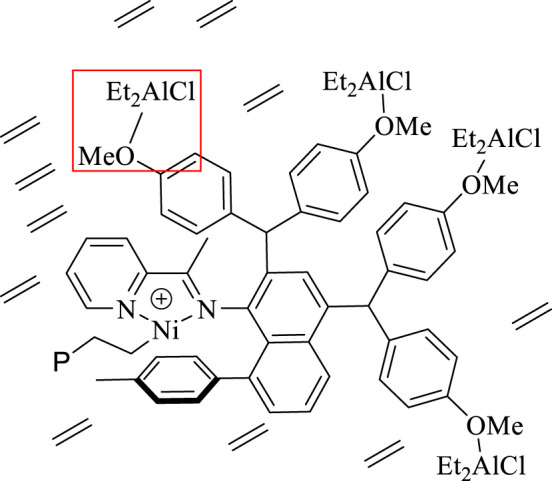
The interaction of OMe with the co-catalyst (Et_2_AlCl) to form a greater axial steric hindrance.

Upon *in-situ* activation with 200 equivalents of Et_2_AlCl, all the Ni(II) complexes exhibited moderate activities (level of 10^5^ g mol^−1^·h^−1^) and yielded high molecular weight (level of 100 kg/mol) polyethylene with high branching densities (57-90/1000 C) and low melting points (-6–53°C) in ethylene polymerization (Table 1; Figures 3, 4). The polymerization activities of these Ni(II) complexes remained almost unchanged with the increase of temperature (Figure 3A). As the ethylene pressure decreased (from 6 to 3 atm and 1 atm), both the polymerization activity and the molecular weight of the resulting polyethylene declined, and the decrease in polymerization activity is more pronounced (Table 1, entries 1 vs 17-18). Similar to the reported nickel-catalyzed ethylene polymerization systems (Zhang et al., 2013), chain termination is mainly based on the pathway of synergistic transfer of polymer chains to monomers. Amidst these five nickel complexes, **Ni4** containing electron withdrawing fluorine and **Ni5** composed of dibenzosuberyl substituent exhibited relatively higher catalytic activity than the others. Probably, a weaker interaction between metal and fluorinated aryl group for **Ni4** and the stronger catalyst thermal stability originating from the proper aryl orientation for **Ni5** may contribute to the better catalytic activities. As opposed to the fact that the pyridine-imine catalysts usually generate low-molecular-weight polyethylene or copolymers in ethylene (co)polymerization due to the unilateral axial steric structure of the pyridine-imine ligand, all these Ni(II) complexes in our case yielded polyethylene with high molecular weight (level of 100 kg/mol), one or two orders of magnitude higher than those obtained in most reported pyridine-imine systems (Figure 5). (Laine et al., 1999; Meneghetti et al., 1999; Laine et al., 2000; Bianchini et al., 2010; Sun et al., 2012; Yue et al., 2014; Sun et al., 2015a; Sun et al., 2015b; Huang et al., 2015; Chen et al., 2016; Huang et al., 2016; Guo et al., 2019; Wang et al., 2020b; Fan et al., 2021; Li and Dai, 2021; Peng et al., 2021; Yan et al., 2022) This is mainly attributed to the synergistic effect of 8-arylnaphthyl and diarylmethyl groups, which form a sandwich-like structure that can effectively retard chain transfer during polymerization. In particular, **Ni5** is capable of generating the highest molecular weight of polyethylene among these catalysts (Figure 2B). An explanation is that the ring structure in the dibenzosuberyl substituent that drives the aryl group closer to the axial position of the metal center. This allows the conversion of the catalyst structure from a semi-sandwich to a full-sandwich structure (Figure 1). It is worth noting that **Ni3** also produced higher molecular weight polyethylene than other catalysts of the same type (**Ni1**, **Ni2** and **Ni4**). This may be due to the interaction of methoxy with the co-catalyst (Et_2_AlCl) to form a greater axial steric hindrance, which can more effectively retard the chain transfer during polymerization process (Figure 6).

**TABLE 1:** Effect of catalysts and temperatures on ethylene polymerization.^a^

Ent	Precat	*T*/^o^c	Yield/g	Act.^*b*^	*M*_n_ (10^4^)^*c*^	*M*_w_/*M*_n_^*c*^	*B* ^*d*^	*T*_m_/(^o^C)^*e*^
1	**Ni1**	30	0.24	2.4	13.71	1.24	68	28
2	**Ni1**	50	0.32	3.2	14.20	1.30	71	21
3	**Ni1**	70	0.29	2.9	15.81	3.29	75	10
4	**Ni2**	30	0.27	2.7	15.39	2.31	74	20
5	**Ni2**	50	0.31	3.1	13.99	1.29	75	17
6	**Ni2**	70	0.29	2.9	13.93	1.59	79	8
7	**Ni3**	30	0.28	2.8	17.36	1.29	57	52
8	**Ni3**	50	0.32	3.2	17.13	1.38	59	53
9	**Ni3**	70	0.31	3.1	17.22	1.54	61	48
10	**Ni4**	30	0.34	3.4	12.86	1.23	68	29
11	**Ni4**	50	0.36	3.6	13.62	1.32	69	29
12	**Ni4**	70	0.38	3.8	14.00	1.60	72	23
13	**Ni5**	30	0.34	3.4	20.22	1.85	87	-4
14	**Ni5**	50	0.36	3.6	19.76	1.61	88	-6
15	**Ni5**	70	0.32	3.2	19.72	1.61	90	-6
16	**Pd5**	30	trace	-	-	-	-	-
17^*f*^	**Ni1**	30	0.12	1.2	10.21	1.43	-	-
18^*g*^	**Ni1**	30	0.03	0.3	7.52	1.56	-	-

^*a*^Conditions: Ni(II) complexes (2 *μ*mol) or Pd(II) complex (10 *μ*mol), 200 eq. Et_2_AlCl, 1 ml of CH_2_Cl_2_, 20 ml toluene, polymerization time (30 min), 6 atm. ^*b*^Activity (Act.) = 10^5^ g/(mol Nih). ^*c*^Determined by GPC in 1,2,4-trichlorobenzene at 150 °C vs polystyrene standards. ^*d*^ *B* = branches per 1,000 carbons, determined by ^1^H NMR spectroscopy, B = 1,000 × 2(I_CH3_)/3(I_CH2+CH_ + I_CH3_). ^*e*^Determined by differential scanning calorimetry (DSC), broad peak.

**FIGURE 7:**
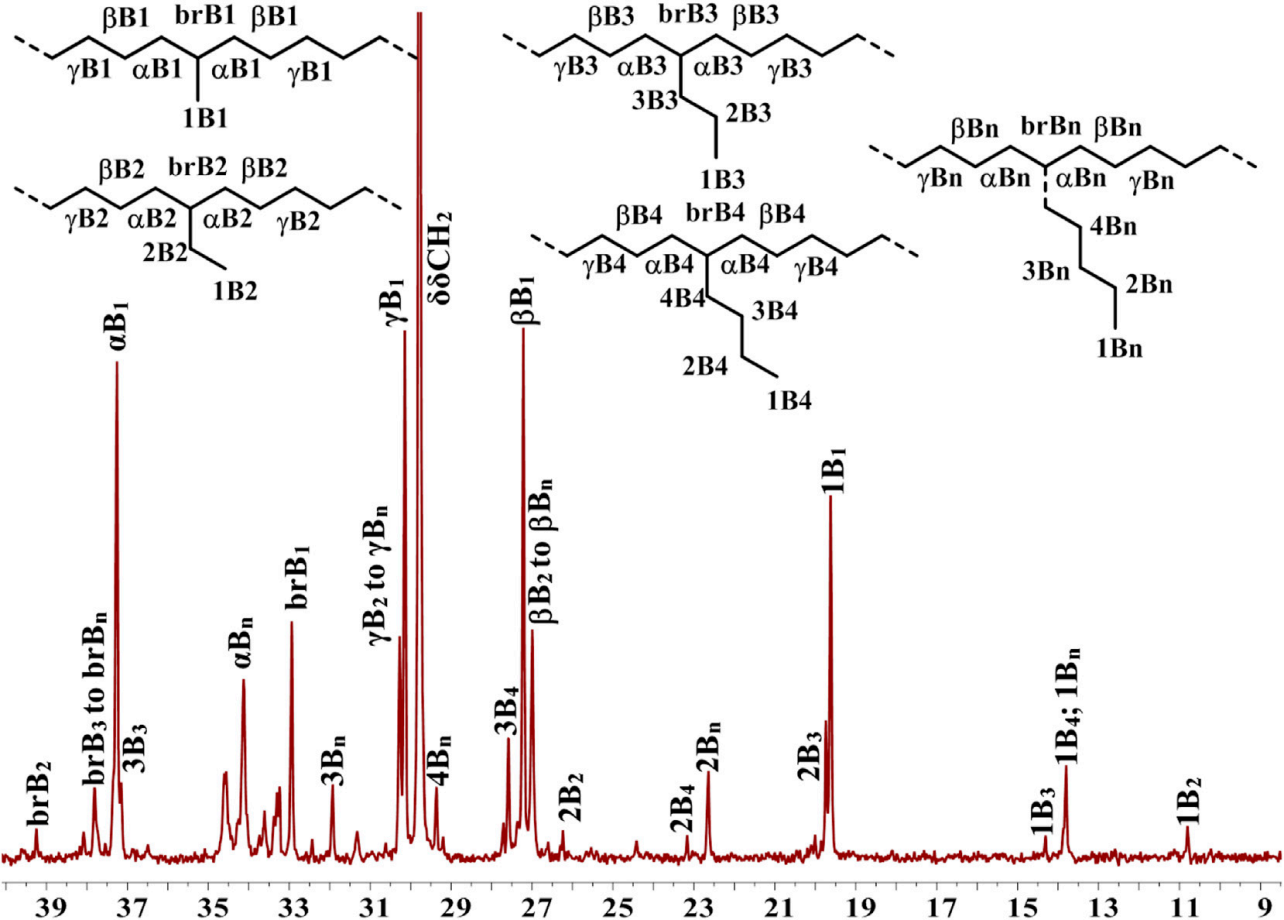
Detailed analysis of ^13^C NMR spectrum of branched polyethylene obtained by using **Ni5** at 70°C (Table 1, entry 15). Assignments are numbered according to ref. 49-51. Branches are labeled as xBy, where y is the branch length and x is the carbon, starting from the methyl end with 1. The methine groups for the different branch lengths are labeled with brBy.

**SCHEME 2:**
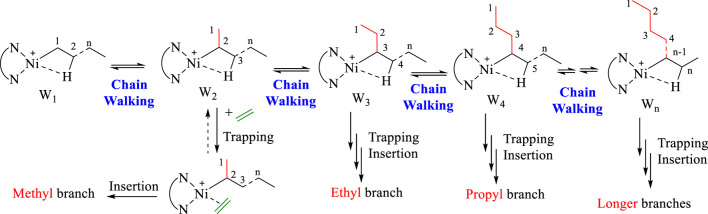
Proposed mechanism for chain walking in pyridine-imine Ni(II) catalytic system.

In addition, **Ni5** yielded the polyethylene with the highest branching density and lowest melting point while **Ni3** produced the polyethylene with the lowest branching density and highest melting point (Figure 2C). As elaborated in previous reports, the sandwich structure of **Ni5** facilitates chain walking processes, thus yielding highly branched polyethylene. (Zhang et al., 2013). By contrast, the interaction of methoxy with the co-catalyst (Et_2_AlCl) in **Ni3** forms a large axial steric hindrance that may disfavor chain walking. The proximity interaction of the co-catalyst with β-H may also be one factor contributing to lowered branching density. (Ma et al., 2021; Li et al., 2020). The microstructure of a representative polyethylene product (Table 1, entry 15) was revealed by ^13^C NMR analysis (Figure 7). (Randall, 1989; Galland et al., 1999; Cotts et al., 2000) The ^13^C NMR analysis suggests that the obtained polyethylene consists of methyl branches, ethyl branches, n-propyl branches and long chain branches formation with chain walking mechanism (Scheme 2). Among them, methyl branches and long chain branches account for the majority of all the branches. This indicates that **Ni5** with a sandwich structure is capable of generating polyethylene with randomly branches distribution in which methyl and long chain branching dominate, further demonstrating its strong chain walking ability. Further comparison with the ^13^C NMR analysis of the polyethylene yielded with **Ni1** and **Ni3** at 70°C, the hybrid “sandwich” structure of **Ni5** facilitates access to a higher percentage of long chain branching (Supplementary Table S1, Supplementary Figure S12).

## Conclusion

In summary, a series of “semi-sandwich” and “sandwich” type pyridine-imine Ni(II) complexes bearing diarylmethyl or dibenzosuberyl groups and 8-aryl-naphthyl substituent were synthesized and characterized. The Ni(II) complexes exhibited moderate activities (level of 10^5^ g/(molh)) and generated highly branched (57-90/1000 C) polyethylene with high molecular weights (level of 10^5^ g/mol) in ethylene polymerization. Moreover, the “full-sandwich” Ni(II) complex containing 8-arylnaphthyl and dibenzosuberyl substituents yielded higher molecular weight polyethylene with higher branching density than those from “semi-sandwich” Ni(II) complexes bearing 8-arylnaphthyl and diarylmethyl groups. In addition, the remote non-conjugated electronic substituents in diarylmethyl groups of the Ni(II) system also have an effect on the ethylene polymerization.

## Data Availability

The original contributions presented in the study are included in the article/Supplementary Material, further inquiries can be directed to the corresponding authors.
