# Supplementary material for: Synthesis of High-Molecular-Weight Branched Polyethylene Using a Hybrid “Sandwich” Pyridine-Imine Ni(II) Catalyst
Source: Front Chem. 2022 May 4;10:886888. doi: 10.3389/fchem.2022.886888 (PMC9114440; doi:10.3389/fchem.2022.886888)
Supplement: Supplementary file 1 [file DataSheet1.docx]

**Synthesis of High-Molecular-Weight Branched Polyethylene Using a Hybrid “Sandwich” Pyridine-Imine Ni(II) Catalyst**

You Ge^†^ *^a,b^*, Qi Cai^†a^, Yuyin Wang *^c^*, Jiangang Gao**^a^*, Yue Chi**^c^*, Shengyu Dai* *^a,b^*

*^a^School of Chemical and Environmental Engineering, Anhui Polytechnic University, Wuhu, Anhui 241000, China.*

*^b^Institutes of Physical Science and Information Technology, Key Laboratory of Structure and Functional Regulation of Hybrid Materials of Ministry of Education, Anhui University, Hefei, Anhui, 230601, China.*

*^c^Key Laboratory of Advanced Structural Materials of Ministry of Education, College of Material Science and Engineering, College of Chemistry and Life Science, Advanced Institute of Materials Science, Changchun University of Technology, Changchun 130012, China*

^†^The first two authors are equal first authors.

**1. Experimental Sections**

**1.1 General Considerations**

All chemicals were commercially sourced, except those whose synthesis is described. All experiments were carried out under a dry nitrogen atmosphere using standard Schlenk techniques or in a glove-box. Deuterated solvents used for NMR were dried and distilled prior to use. ^1^H and ^13^C NMR spectra were recorded by a JNM-ECZ400R or JNM-ECZ600R spectrometer at ambient temperature unless otherwise stated. The chemical shifts of the ^1^H and ^13^C NMR spectra were referenced to the residual solvent; Coupling constants are in Hz. Mass spectra were obtained by the Analytical Center of Anhui University. Elemental analysis was performed by the Analytical Center of Anhui University. X-ray Diffraction data were collected at 293(2) K on a Bruker Smart CCD area detector with graphite-monochromated Mo K^α^ radiation (λ = 0.71073 Å). Molecular weight and molecular weight distribution of the polymers were determined by gel permeation chromatography (GPC) with a PL 210 equipped with one Shodex AT-803S and two Shodex AT-806MS columns at 150 ^o^C using trichlorobenzene as a solvent and calibrated with polystyrene standards. Differential scanning calorimetry (DSC). DSC was performed by a DSC Q25 from TA Instruments. Samples were quickly heated to 150°C and kept for 5 min to remove thermal history, then cooled to -50°C at a rate of 10 K/min, and finally reheated to 150°C at the same rate under a nitrogen flow (50 mL/min). The maximum endotherm points (heating scan) were taken as the melting temperature (*T*_m_).

**1.2 Procedure for the Synthesis of Nickel Complexes Ni1-Ni5.**

Complexes **Ni1-Ni5** were synthesized by the reaction of 1 equiv. of NiBr_2_(DME) with the corresponding ligands in methylene chloride. The corresponding ligand (0.2 mmol) was added in 5 mL of methylene chloride in a Schlenk tube under a nitrogen atmosphere. NiBr_2_(DME) (0.2 mmol, 62 mg) was added to the above solution. The resulting mixture was stirred at room temperature overnight. The solvent was evaporated under reduced pressure to afford a solid. The product was washed with 4 × 5 mL diethyl ether and dried under vacuum.

**Ni1:** (167 mg, 94%). MALDI-TOF-MS (m/z): calcd for C_50_H_40_BrN_2_Ni: 807.17, Found, 807.25, [M-Br]^+^. Elemental analysis: calc. for C_50_H_40_Br_2_N_2_Ni: C, 67.68; H, 4.54; N, 3.16. Found: C, 67.54; H, 4.38; N, 3.24.

**Ni2:** (172 mg, 91%). MALDI-TOF-MS (m/z): calcd for C_54_H_48_BrN_2_Ni: 863.23, Found, 863.27, [M-Br]^+^. Elemental analysis: calc. for C_54_H_48_Br_2_N_2_Ni: C, 68.74; H, 5.13; N, 2.97. Found: C, 68.65; H, 5.31; N, 2.88.

**Ni3:** (163 mg, 81%). MALDI-TOF-MS (m/z): calcd for C_54_H_48_BrN_2_NiO_4_: 927.21, Found, 927.21, [M-Br]^+^. Elemental analysis: calc. for C_54_H_48_Br_2_N_2_NiO_4_: C, 64.38; H, 4.80; N, 2.78. Found: C, 64.25; H, 4.68; N, 2.84.

**Ni4:** (167 mg, 87%). MALDI-TOF-MS (m/z): calcd for C_50_H_36_BrF_4_N_2_Ni: 879.13, Found, 879.19, [M-Br]^+^. Elemental analysis: calc. for C_50_H_36_Br_2_F_4_N_2_Ni: C, 62.60; H, 3.78; N, 2.92. Found: C, 62.42; H, 3.84; N, 2.89.

**Ni5:** (158 mg, 84%). MALDI-TOF-MS (m/z): calcd for C_54_H_44_BrN_2_Ni: 859.20, Found, 859.23, [M-Br]^+^. Elemental analysis: calc. for C_54_H_44_Br_2_N_2_Ni: C, 69.04; H, 4.72; N, 2.98. Found: C, 68.99; H, 4.57; N, 2.89.

**1.3 Procedure for the Synthesis of Palladium Complex Pd5.**

A mixture of the ligand (0.2 mmol), (COD)PdCl_2_ (0.2 mmol) in CH_2_Cl_2_ (10 mL) was stirred for 24 h at room temperature. During stirring, the color of the solution was deepening. At the end of the reaction, the solvent was partially evaporated under reduced pressure. The remaining mixture was diluted with Et_2_O (20 mL). The resulting yellow solid was collected by filtration, dried in vacuum.

**Pd5:** (153 mg, 85%). ^1^H NMR (600 MHz, Chloroform-*d*) δ 9.34 (d, *J* = 5.4 Hz, 1H, Ar-*H*), 8.48 (d, *J* = 8.6 Hz, 1H, Ar-*H*), 8.30 – 8.13 (m, 1H, Ar-*H*), 7.93 (t, *J* = 7.4 Hz, 1H, Ar-*H*), 7.76 (d, *J* = 7.5 Hz, 1H, Ar-*H*), 7.64 (t, *J* = 7.8 Hz, 1H, Ar-*H*), 7.59 (d, *J* = 7.5 Hz, 1H, Ar-*H*), 7.56 – 7.48 (m, 1H, Ar-*H*), 7.36 (s, 1H, Ar-*H*), 7.36 – 7.32 (m, 1H, Ar-*H*), 7.23 – 7.02 (m, 10H, Ar-*H*), 6.91 (d, *J* = 7.4 Hz, 1H, Ar-*H*), 6.86 (d, *J* = 7.5 Hz, 1H, Ar-*H*), 6.81 – 6.75 (m, 3H, Ar-*H*), 6.72 (t, *J* = 7.3 Hz, 1H, Ar-*H*), 6.50 (s, 1H, C*H*Ar_3_), 6.35 (d, *J* = 7.4 Hz, 1H, Ar-*H*), 6.07 (t, *J* = 7.3 Hz, 1H, Ar-*H*), 5.79 (s, 1H, C*H*Ar_3_), 2.95 (dtd, *J* = 32.3, 14.1, 4.6 Hz, 2H, C*H*_2_C*H*_2_), 2.67 – 2.53 (m, 2H, C*H*_2_C*H*_2_), 2.27 (d, *J* = 17.4 Hz, 2H, C*H*_2_C*H*_2_), 2.21 – 2.13 (m, 1H, C*H*_2_C*H*_2_), 2.11 – 2.02 (m, 1H, C*H*_2_C*H*_2_), 1.91 (s, 3H, Ar-C*H*_3_), 0.80 (s, 3H, C(C*H*_3_)=N). ^13^C NMR (151 MHz, Chloroform-*d*) δ 178.64 (*C*(CH_3_)=N), 156.52, 150.35, 142.25, 141.63, 141.49, 141.06, 141.05, 140.12, 139.21, 138.72, 138.62, 138.18, 137.62, 137.55, 137.15, 136.01, 133.14, 132.53, 132.49, 132.43, 132.17, 132.06, 131.99, 131.92, 131.50, 131.40, 130.44, 130.34, 130.29, 129.02, 128.08, 127.67, 127.40, 127.12, 126.97, 126.89, 126.51, 126.12, 125.99, 125.89, 125.68, 125.42, 125.30, 125.09, 124.88, 57.76 (*C*HAr_3_), 55.64 (*C*HAr_3_), 32.62 (*C*H_2_*C*H_2_), 32.45 (*C*H_2_*C*H_2_), 30.10 (*C*H_2_*C*H_2_), 29.39 (*C*H_2_*C*H_2_), 21.03 (Ar-*C*H_3_), 18.03 (C(*C*H_3_)=N). ESI-MS (m/z): calcd for C_54_H_44_Cl_2_N_2_PdNa^+^: 918.18, Found, 918.20, [M+Na] ^+^. Elemental analysis: calc. for C_54_H_44_Cl_2_N_2_Pd: C, 72.20; H, 4.94; N, 3.12. Found: C, 72.12; H, 4.87; N, 3.24.

**1.4 A General Procedure for the Homopolymerization of Ethylene.**

In a typical experiment, a 350 mL thick-walled pressure glass vessel was firstly dried at 90 °C under vacuum for at least 1 h. The vessel was then connected with a high pressure gas line and adjusted to the desired polymerization temperature. 20 mL of toluene and the desired amount Et_2_AlCl was added to the vessel under ethylene atmosphere, then the desired amount of catalyst in 1 mL of CH_2_Cl_2_ was injected into the polymerization system via syringe. With a rapid stirring, the vessel was pressurized and maintained at 6 atm of ethylene. After 30 min, the pressure vessel was vented and the polymer was precipitated in ethanol, filtered and dried at 50 °C for at least 24 h under vacuum.

**2. Spectra Data**

**2.1 ^1^H, ^13^C NMR of the Synthetic Compounds.**


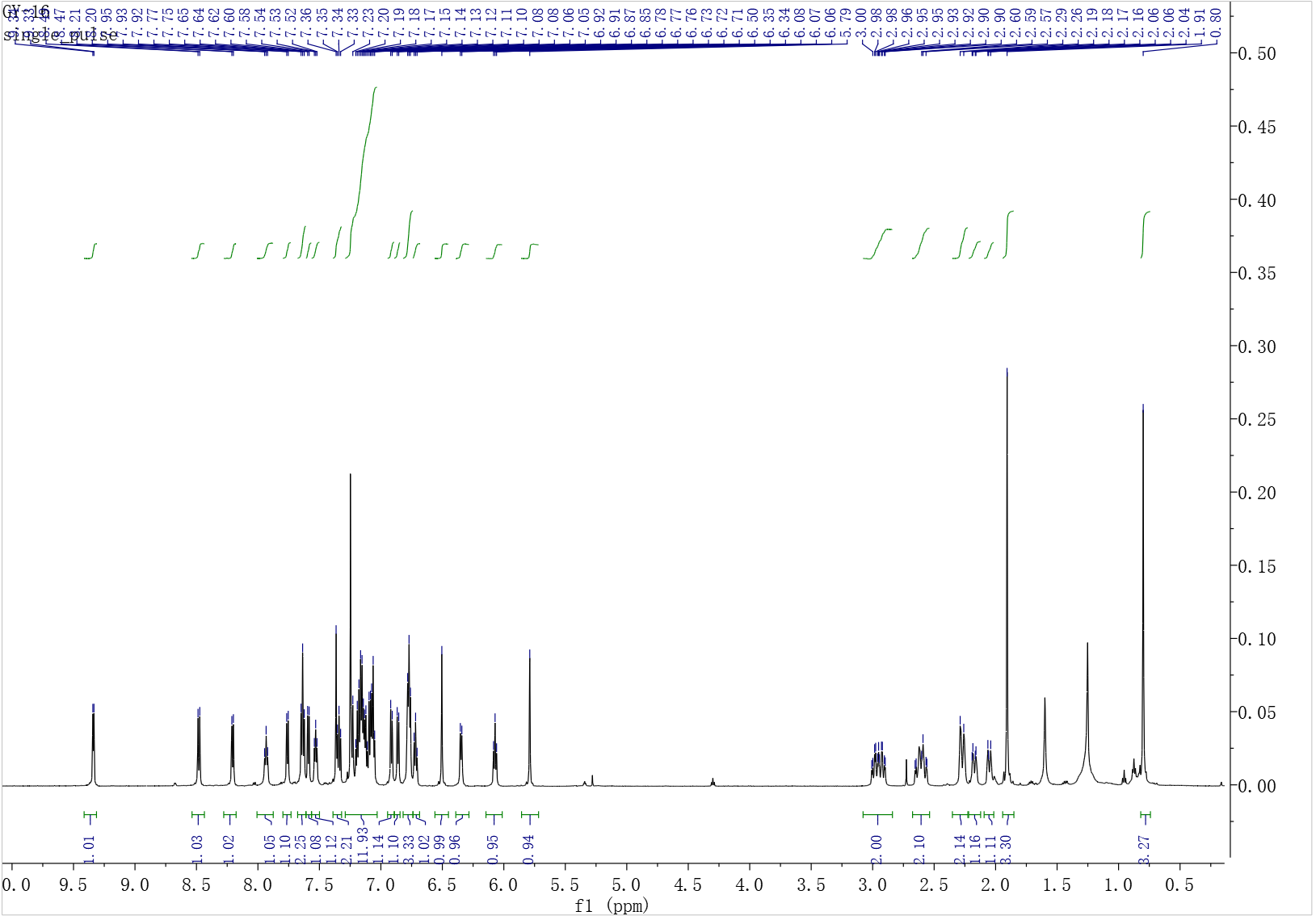


**Figure S1.** ^1^H NMR spectrum of **Pd5** in CDCl_3_.

**
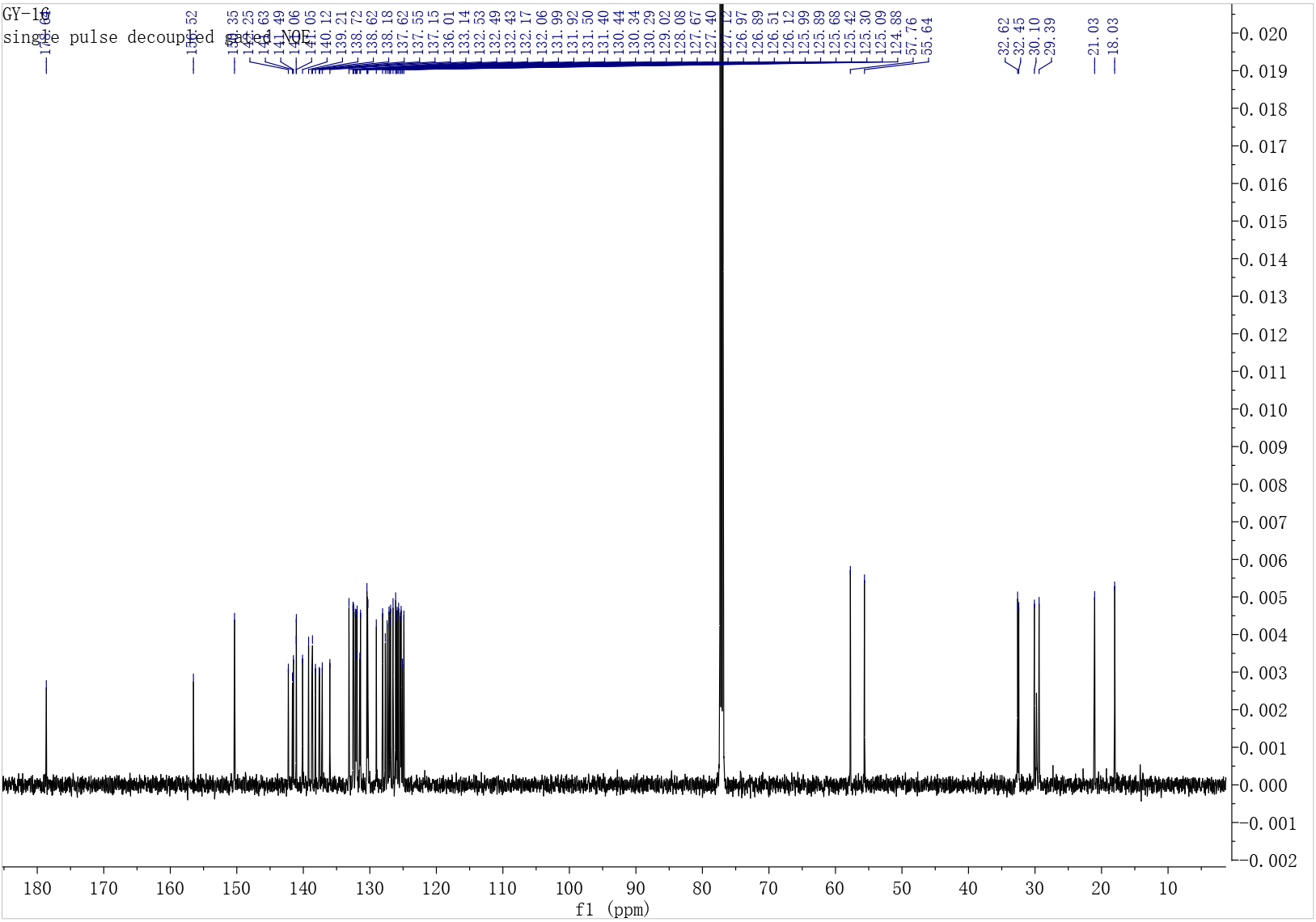
**

**Figure S2.** ^13^C NMR spectrum of **Pd5** in CDCl_3_.

**2.2 MS of Complexes Ni1-Ni5 and Pd5**

**Figure S3.** MALDI-TOF MS of **Ni1** in CDCl_3._

**Figure S4.** MALDI-TOF MS of **Ni2** in CDCl_3._

**Figure S5.** MALDI-TOF MS of **Ni3** in CDCl_3._

**Figure S6.** MALDI-TOF MS of **Ni4** in CDCl_3._

**Figure S7.** MALDI-TOF MS of **Ni5** in CDCl_3._

**Figure S8.** ESI-MS of **Pd5** in CDCl_3._

**2.3 ^1^H NMR of Representative Polymers.**

**
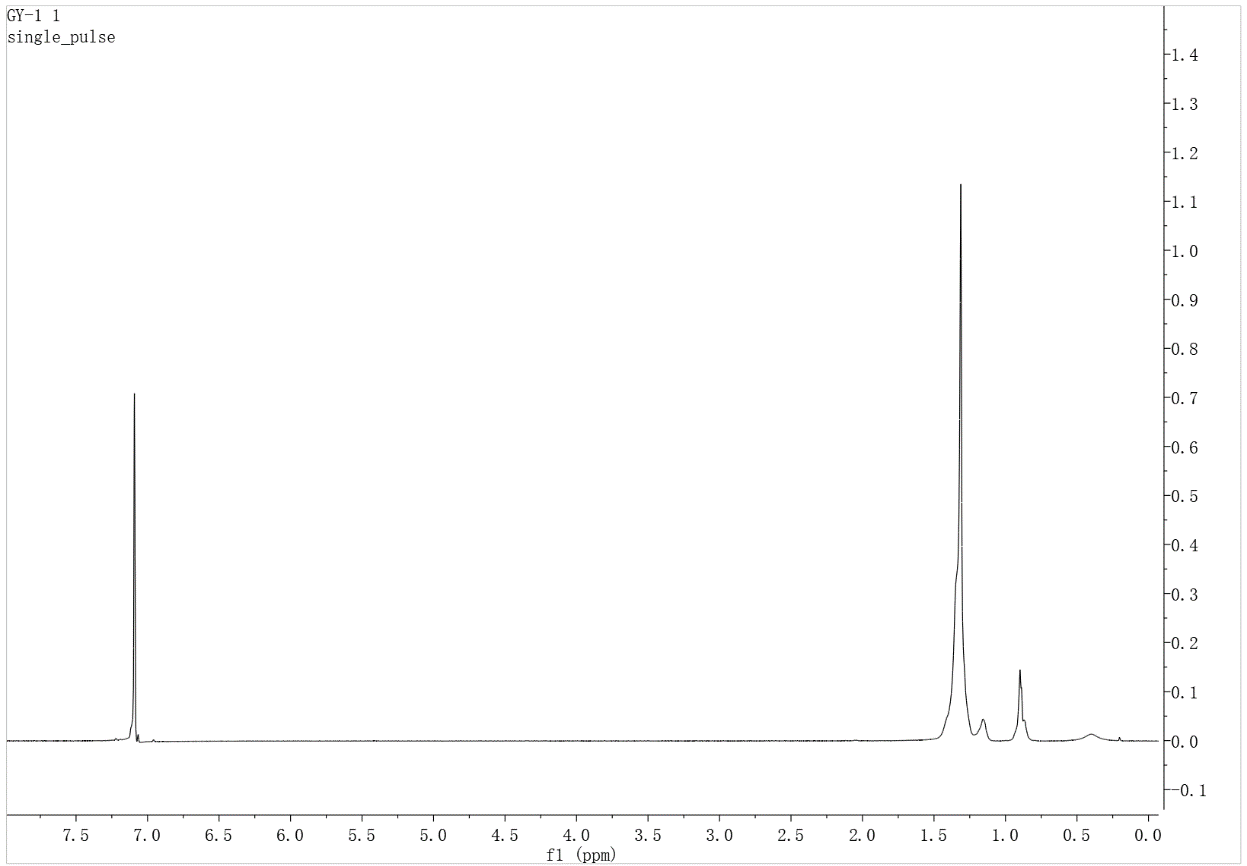
**

**Figure S9**. ^1^H NMR spectrum of the polymer from table 1, entry 1 (C_6_D_6_, 70 °C).

**
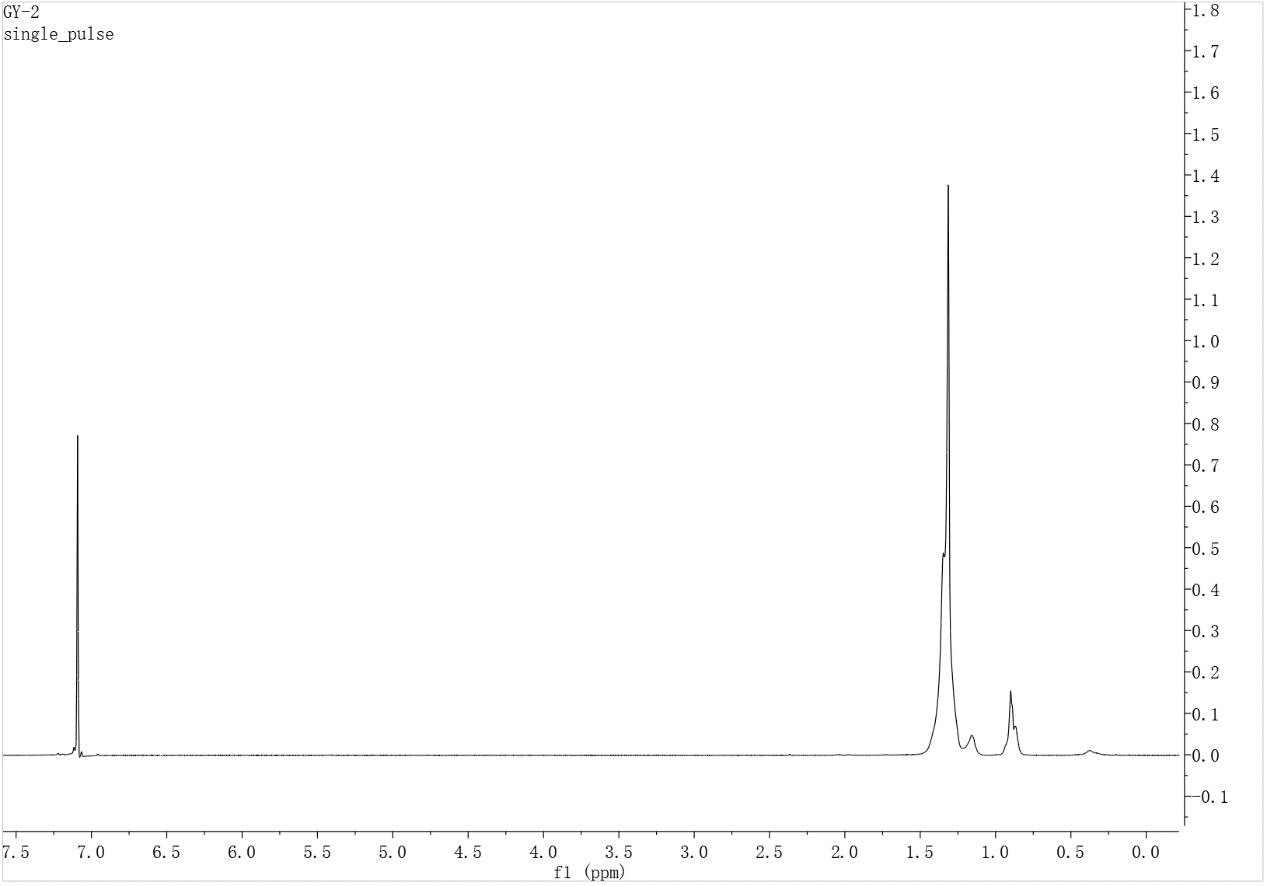
**

**Figure S10**. ^1^H NMR spectrum of the polymer from table 1, entry 2 (C_6_D_6_, 70 °C).

**
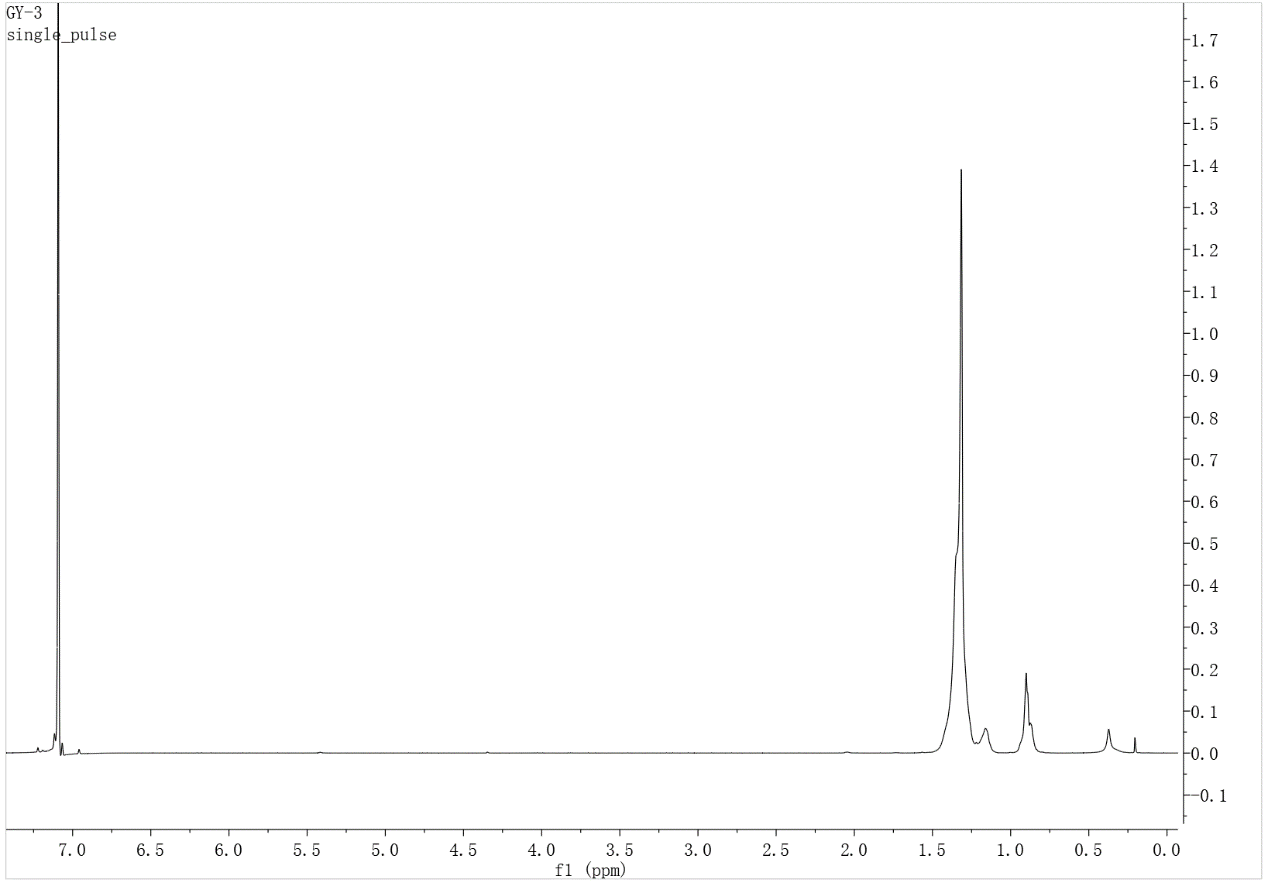
**

**Figure S11**. ^1^H NMR spectrum of the polymer from table 1, entry 3 (CDCl_3_, 20 °C).


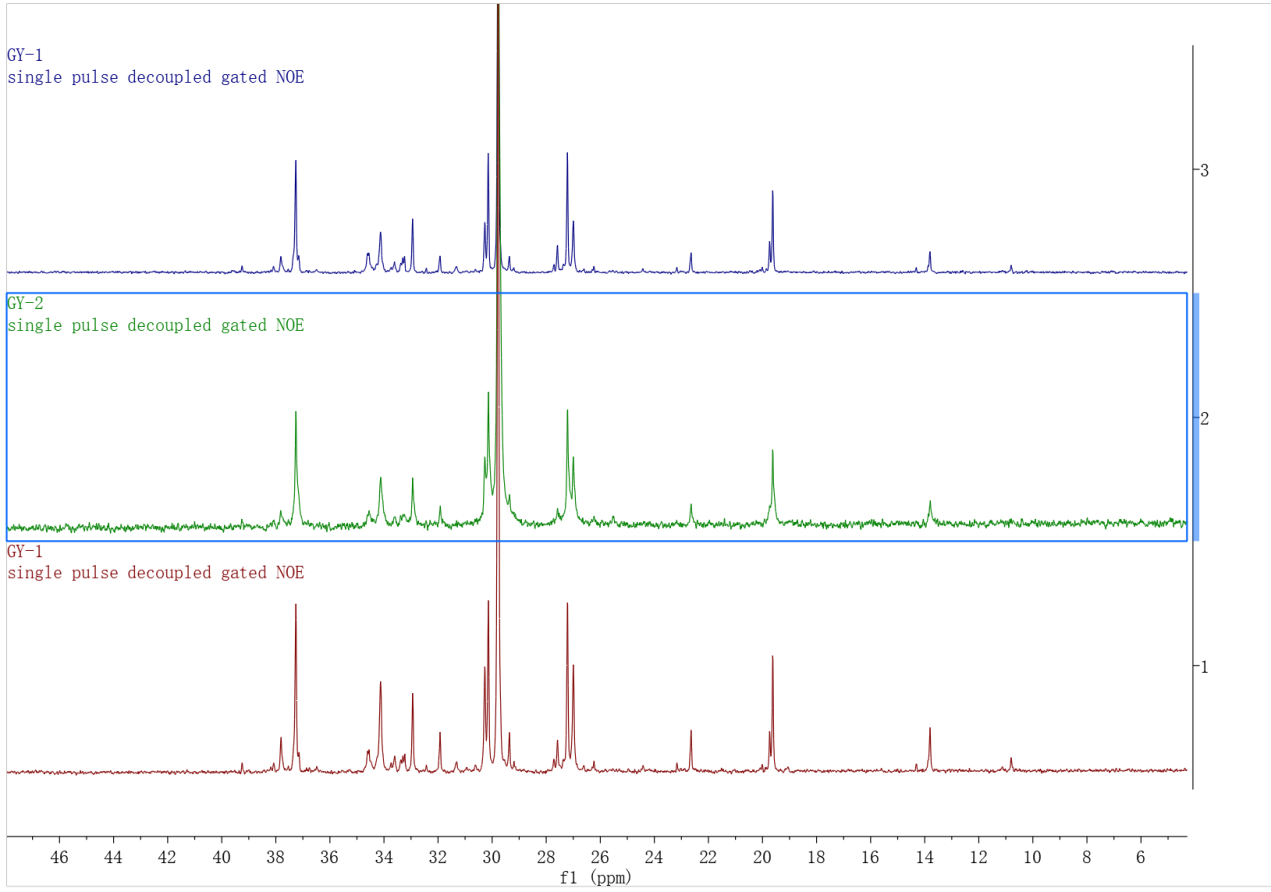


**Figure S12.** ^13^C NMR spectrum of the polyethylene yielded with **Ni1**, **Ni3** and **Ni5** at 70 ^o^C from Table 1, entries 3, 9 and 15.

**Table S1.** Microstructure analysis with fractional amounts of different branch lengths*^a^*

| Ent. | PE | Branches  /1000 C | methyl*^a^*  (%) | ethyl*^a^*  (%) | propyl*^a^*  (%) | C_4+_*^a^*  (%) |
| --- | --- | --- | --- | --- | --- | --- |
| 1*^b^* | **Ni1-70** | 75 | 74 | 6 | 2 | 18 |
| 2*^b^* | **Ni3-70** | 61 | 75 | 5 | 3 | 17 |
| 3*^b^* | **Ni5-70** | 90 | 71 | 5 | 3 | 21 |

*^a^*Percentages of different branch lengths can be calculated from the relative intensity ratios of the methyl (1B_1_, 1B_2_, 1B_3_, 1B_n_) signals of the respective branches in the ^13^C NMR spectra. *^b^*PE from Table 1, entries 3, 9 and 15.

**2.4 DSC and GPC of Representative Polymers.**

**Figure S13**. DSC of the polymer from table 1, entry 4.

**Figure S14**. GPC of the polymer from table 1, entry 5.

**Figure S15**. GPC of the polymer from table 1, entry 6.

**
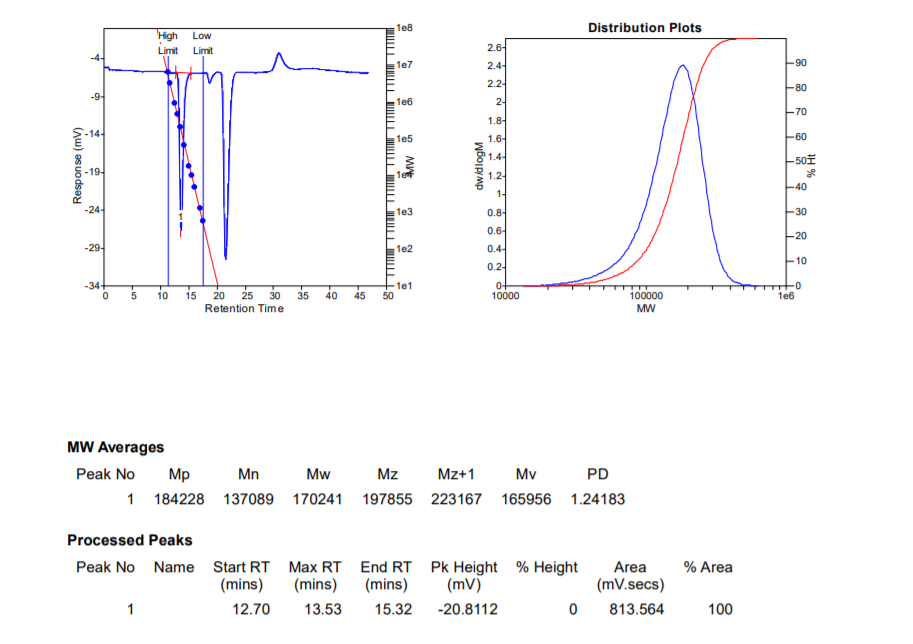
**

**Figure S16**. GPC of the polymer from table 1, entry 1.

**
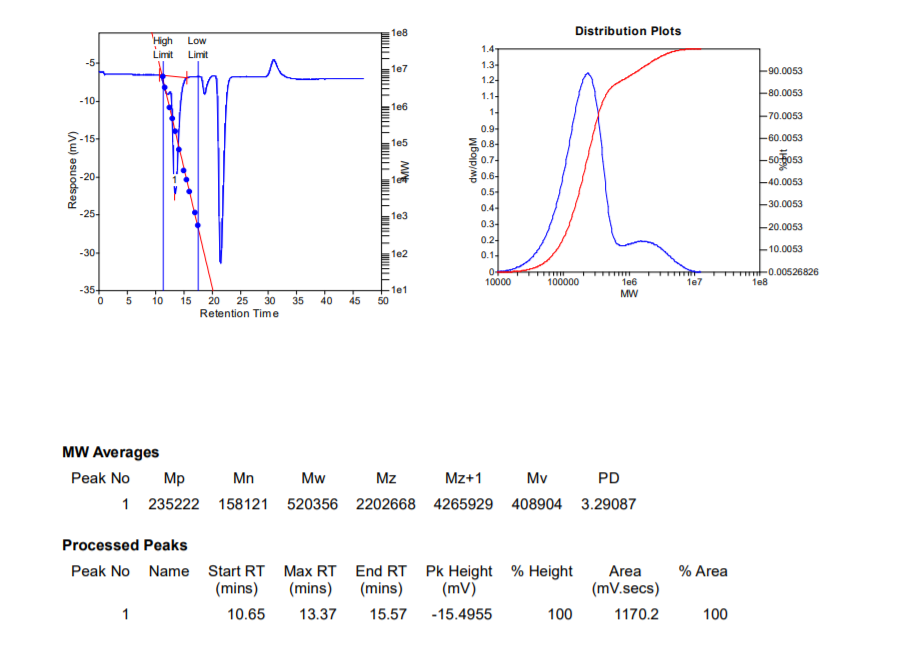
**

**Figure S17**. GPC of the polymer from table 1, entry 3.

**3. X**-**ray Crystallography**


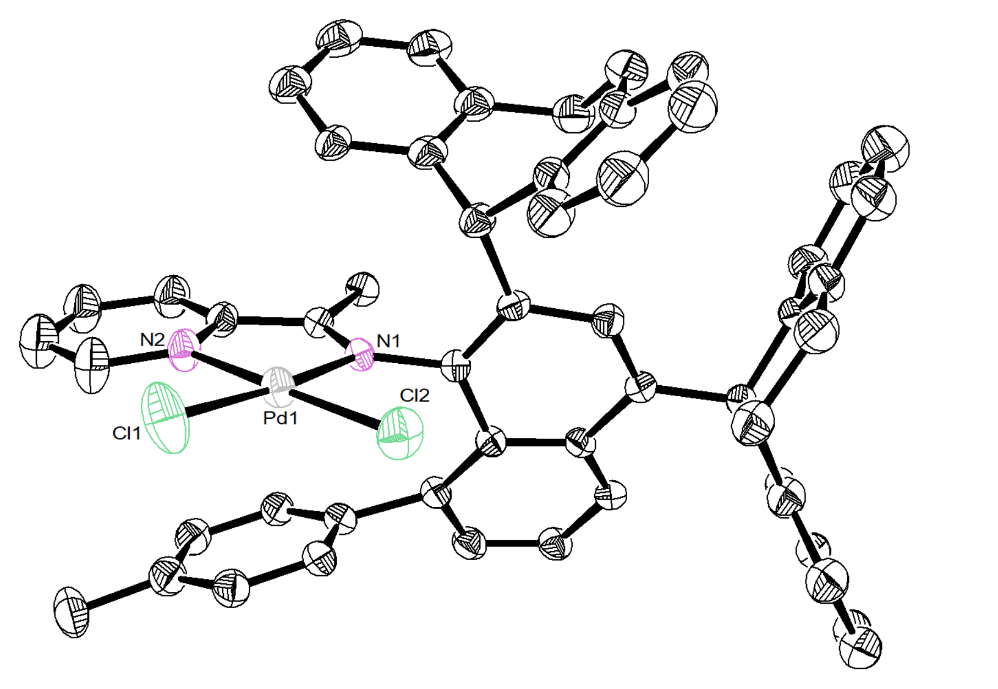


| **Table S2 Crystal data and structure refinement for Pd5.** | |
| --- | --- |
| Identification code | **Pd5** |
| Empirical formula | C55 H46 Cl4 N2 Pd |
| Formula weight | 983.14 |
| Temperature/K | 293(2) |
| Crystal system | orthorhombic |
| Space group | P2_1_2_1_2_1_ |
| a/Å | 10.5146(7) |
| b/Å | 16.1122(11) |
| c/Å | 26.495(2) |
| α/° | 90 |
| β/° | 90 |
| γ/° | 90 |
| Volume/Å^3^ | 4488.6(6) |
| Z | 4 |
| ρ_calc_g/cm^3^ | 1.455 |
| μ/mm^‑1^ | 5.835 |
| F(000) | 2016.0 |
| Crystal size/mm^3^ | 0.03 x 0.02 x 0.01 |
| Radiation | CuKα (λ = 1.54186) |
| 2Θ range for data collection/° | 8.642 to 124.982 |
| Index ranges | -12<=h<=8, -18<=k<=13, -30<=l<=30 |
| Reflections collected | 17928 |
| Independent reflections | 7043[R_int_ = 0.0778, R_sigma_ = 0.1055] |
| Data/restraints/parameters | 7043 / 696 / 561 |
| Goodness-of-fit on F^2^ | 0.936 |
| Final R indexes [I>=2σ (I)] | R_1_ = 0.0642, wR_2_ = 0.1428 |
| Final R indexes [all data] | R_1_ = 0.1053, wR_2_ = 0.1625 |
| Largest diff. peak/hole / e Å^-3^ | 0.88 / -0.34 |
| Flack parameter | 0.016(13) |
